# Oral Exposure to Genistein during Conception and Lactation Period Affects the Testicular Development of Male Offspring Mice

**DOI:** 10.3390/ani10030377

**Published:** 2020-02-26

**Authors:** Zhicheng Shi, Zengpeng Lv, Chenhui Hu, Qing Zhang, Zhe Wang, Enayatullah Hamdard, Hongjian Dai, Sheeraz Mustafa, Fangxiong Shi

**Affiliations:** College of Animal Science and Technology, Nanjing Agricultural University, No. 1 Weigang, Nanjing 210095, China; 2017105023@njau.edu.cn (Z.S.); lvzengpeng@njau.edu.cn (Z.L.); 2018805105@njau.edu.cn (C.H.); 15116416@njau.edu.cn (Q.Z.); 2018105020@njau.edu.cn (Z.W.); 2017105117@njau.edu.cn (E.H.); 2018105021@njau.edu.cn (H.D.); sheerazmustafa786@gmail.com (S.M.)

**Keywords:** genistein, testes, spermatid, infertility

## Abstract

**Simple Summary:**

Spermatogenesis and hormones secretions are important life-threating and complicated process, which can be affected by environmental estrogens. Genistein, a type of isoflavones, widely exists in the soybean products diet, which exerts a controversial role in reproductive regulation for its special structures or functions. The results of the study revealed that low-dose genistein treatment increased the level of testosterone in the mice serum, and positively regulated expression of spermatogenesis-related genes, which enhanced spermatogenesis and testicular development. However, High-dose genistein treatment induced apoptosis of germ cells and inhibited proliferation of germ cells during spermatogenesis. Reproductive alterations in the structures and functions of testis were dose-dependent in different genistein treatments.

**Abstract:**

Sexual hormones are essential for the process of spermatogenesis in the testis. However, the effect of maternal genistein (GEN) on the pups’ testicular development remain-unclear. Our present study evaluated the effects of supplementing GEN for parental and offspring mice on the reproductive function and growth performance of the male pups. Mothers during gestation and lactation period were assigned to a control diet (CON group), low dose GEN (LGE group) diet (control diet +40 mg/kg GEN), and high dose of GEN (HGE group) diet (control diet +800 mg/kg GEN). Their male offspring underwent the same treatment of GEN after weaning. LGE treatment (40 mg/kg GEN) significantly increased body weights (*p* < 0.001), testes weights (*p* < 0.05), diameters of seminiferous tubule (*p* < 0.001) and heights of seminiferous epithelium (*p* < 0.05) of offspring mice. LGE treatment also increased serum testosterone (T) levels and spermatogenesis scoring (*p* < 0.05). However, HGE treatment (800mg/kg GEN) significantly decreased body weights (*p* < 0.001), testes weights (*p* < 0.05) and testis sizes (*p* < 0.001). Furthermore, mRNA expressions of *ESR2* (*p* < 0.05), *CYP19A1* (*p* < 0.001), *SOX9* (*p* < 0.001) and *BRD7* (*p* < 0.001) in testis of mice were increased in the LGE group. Similarly, HGE treatment increased mRNA expressions of *ESR2* (*p* < 0.05) and *CYP19A1* (*p* < 0.001). However, mRNA expressions of *SOX9* and *BRD7* were decreased significantly in the HGE group (*p* < 0.001). Meanwhile, higher ratio apoptotic germ cells and abnormal sperms were detected in the HGE group (*p* < 0.001). In conclusion, exposure to a low dose of GEN during fetal and neonatal life could improve testicular development of offspring mice, whereas, unfavorable adverse effects were induced by a high dose of GEN.

## 1. Introduction

Environmental estrogen, as a type of endocrine disruptors, is currently receiving extensive attention [1,2]. The major class of soy phytoestrogens exert a controversial role in reproductive regulation for its special structures or functions [3,4,5]. Testes (GEN), a type of isoflavones, widely exists in the soybean products diet, which exerts a controversial role in reproductive regulation for its special structures or functions [6]. One previous study reported that GEN could be deposited to the suckling offspring from the pregnant mice [7]. Thus, mice in fetal and neonatal periods could expose to the exogenous estrogen when the mother was fed GEN. Recently, our research revealed that adding a low dose of GEN (40 mg/kg) into the diet of breeder hens and their offspring could improve the growth performance and immune state of the male offspring chicks [8,9]. Similarly, human offspring habitually exposed to their family diet. This is worthy of attention, that approximately 20% of U.S. infants are fed soy formula, and one-third of Americans consume soy products every week [10]. Lager population of Chinese soy consumers was reported because of eating habits [11]. Therefore, it is of great significance for human health to investigate the reproductive function of offspring when pups continuously exposed to GEN from the fetal period to maturity.

GEN caused controversial results, including reproductive disorders and other beneficial effects [12]. It is suggested that GEN injection made adverse effects on estrous cyclicity and ovarian differentiation [6,12,13]. The report that GEN could induce reproductive toxicity in adult and infant animals has been extensively investigated [14]. Whereas, some researchers suggested that isoflavone promoted the maturation of germ cells [15,16,17,18,19]. The proliferation of germ cells was observed when germ cells were maintained in medium containing 1μmol/L GEN [7]. Moreover, orally exposing to isoflavone at 600 mg/kg appears to be beneficial in increasing testicular weight, Sertoli cell area and seminiferous tubule volume [20]. In addition, the previous study revealed that GEN could significantly increase the expression of StAR, which accounts for the increment of spermatogonia number and germ cell layers [21].

Previous studies demonstrated that GEN exposure during mice lactation period could make adverse effects on the reproductive health of pups [22,23,24], even if the metabolic capacity of neonatal mice has not developed completely [25,26]. Moreover, oral exposure to GEN elicited growth inhibition of male pups [27]. As we all know, males are more susceptible than females to endocrine disruption during the fetal period and early postnatal weeks [28]. Montani et al. reported that GEN could pass from the lactating mother to the suckling offspring at more than 500mg/kg and result in compromising fertility [7]. However, the mechanism of continuous exposure to GEN on testicular development in mice from the fetal to puberty period remains unclear.

Estrogen receptors, *ESR1* and *ESR2*, can mediate estrogen action [29]. Aromatase, encoded by *CYP19A1* gene, is a key factor in the biosynthesis of estrogens [30,31]. *SOX9*, a biomarker of the Sertoli cell, is the essential and indispensable regulator for survival and proliferation of germ cells [32]. *BRD7* is also a germ cells maker which widely expressed during testicular development [33]. *SOX9* and *BRD7* are involved in male infertility and spermatogenesis [32]. Furthermore, *BRD7* can induce growth-inhibition and cell cycle delay effects though regulating Ras/MEK/ERK and Rb/E2F pathways [33]. GEN is generally known for its estrogenic function through an ER-mediated mechanism. Meanwhile, GEN is a tissue-specific androgen receptor (AR) modulator and play its role in AR signaling including down-regulating genes expression, such as *CYP19A1* [7,34,35]. Moreover, AMP- activated kinase (AMPK) is present in the seminiferous epithelium and interstitium of the testis, which could be activated by GEN [36,37,38]. Therefore, we speculated that GEN might exert its effects on testis development and spermatogenesis of mice by regulating these genes.

Several lines of evidence have suggested that GEN exerts different effects on the male reproductive system, which depends on the dose and species [21,39]. However, whether there is a dosing limitation for predicting health risks remain unclear. In the current experiment, we supplemented pregnant mice with low- and high- dose dietary GEN continuously from pregnancy to neonatal mice. To clarify the maternal effect of GEN on the testicular functions of male offspring.

## 2. Materials and Methods

### 2.1. Animal Ethics Statement

All procedures for animal handling were conducted under protocols approved by the Animal Welfare Committee of Nanjing Agricultural University, China. All animals were treated compassionately and with regard for alleviation of suffering, and they produce comply with animal care guidelines (Permit number: SYXK (Su) 2019–0036).

### 2.2. Animals and Treatments

The study extended on two generations of CD-1 mice following a parental group of mice that were exposed to GEN feed. Treatment began for two weeks before the mother gives pups. After postpartum, male pups (Post-Natal days 1; PND 1) from the same treatment group were collected together, and randomly standardized to seven male pups per group (n = 7). After weaning, the mother was removed, and treatment continued on the pups until they were 35 days old. Living conditions were under controlled of temperature (21 ± 1 °C), lighting (12/12-hr light/dark cycle). Female mice (F0) were fed with a standard laboratory diet and fresh water ad libitum. Treatment began at pregnancy day PD7 (PD7) of the female mice with a special diet which maintained different dosage of GEN- (LGE: 40 mg; he: 800 mg. GEN is a synthetic product from Kai Meng Co. (Xi An, China) Chemical Plant with 99.8% purity). Also, a group of pregnant mice that fed with standard laboratory diet were labeled as control group. 

### 2.3. Sample Collection

After weighing the body, the pups were cervical dislocated under CO_2_ anesthesia at PND 35. Testis and epididymis were weighted and collected. Blood samples were also collected. The serum was centrifuged at 1600 g for 10 minutes and stored at −20 °C until use for the detection of hormones. Randomly one testis was fixed for histomorphology analysis, and another testis was stored at −80 °C for the analysis of gene expression.

### 2.4. Histomorphology under Light Microscope

Pathological sections were prepared, as described in the previous report [40]. Testicular samples were fixed in 10% formaldehyde solution for 24 h. Fixed tissues were dehydrated in a graded series of alcohol, rendered transparent in xylene, and embedded in paraffin. Sections (4-μm thickness) were continuously sliced and stained with hematoxylin and eosin (HE). The histological structure was then observed under a light microscope *BX51* (OLYMPUS, Tokyo, Japan). Ten seminiferous tubules of the testis from each replicate were evaluated, under 20×, 40×, and 100× magnifications. The diameter of seminiferous tubules and the height of seminiferous epithelial were recorded in micrometers. The diameter of a seminiferous tubule was defined as the shortest distance when the length of the outer edge of the tubule measured by two vertical measuring scales. The seminiferous epithelial height was defined as the shortest distance between the germ cell, which was closest to the center of the lumen and the basement membrane. The methods of measurement were followed by previous study [41]. 

### 2.5. Transmission Electronic Microscopy

Testicular tissues were removed from CON, LGE and HGE group mice. Tissues were fixed overnight with 5% glutaraldehyde (Sigma-Aldrich, Shanghai, China) in 0.1 M phosphate buffer (pH 7.4) and subsequently for 3 h in 1.0% osmium tetroxide. The testis was washed in 0.1 M sodium phosphate buffer and then post fixed three times in 1% osmium tetroxide in 0.1 M sodium phosphate buffer at 4 °C. After repeated washing steps, the tissues were dehydrated with a graded series of acetone and embedded in Epon812, DDSA (dodecenylsuccinic anhydride), MNA (methylnadic anhydride), and DMP30 (dimethylaminomethyl phenol) at 60 °C for 48 h. Semi-thin 1 μ m-thick sections were routinely stained with toluidine blue for light microscopy. Ultrathin 70-nm-thick sections were prepared by Leica EM UC6 (TEOL Ltd, Osaka, Japan) and were contrasted with uranyl acetate and lead citrate and examined using a Jeol1230 Hitachi electron microscope (TEOL Ltd, Osaka, Japan). Digital images were captured using a MegaView III digital camera (Leica microsystem Trading company, Shanghai, China).

### 2.6. Spermatogenesis Analysis

Scoring method of morphology assessment and progression of the germinal epithelium in the seminiferous tubules was carried out according to our evaluation standard. Briefly, the score applies a grade from 0 to 5 to each tubule cross section according to the following criteria: Table 1. Staging method was formed for observation based on previous studies [41,42].

### 2.7. Immunohistochemistry

The tissues treated above were deparaffinized and hydrated via graded xylenes and ethanol, followed by heat-induced anti-gen retrieval. Endogenous peroxidase activity and non-specific binding sites for antibodies were blocked with hydrogen peroxide and bovine serum albumin (BSA) for one and two hours, respectively. The sections were incubated with primary antibody (PCNA dilution 1:150; GB13030; Nanjing Vazyme Biotech, Nanjing, China) overnight at 4 °C. Rabbit IgG-SABC kits (ZB-2301; Boster Biological Technology, Wuhan China) were used to detect the immunoreactivity of these specific proteins (Add rabbit IgG to the section, washed with PBS and add SABC). The immunolabeling was visualized with 0.05% DAB (3, 3’ -diaminobenzidine tetrachlo-ride, Solebo biotech Ltd, Suzhou, China) in PBS for 30 s. Finally, the sections were stained with hematoxylin, covered with coverslips and then viewed under Olympus *BX51* photomicroscope (OLYMPUS, Tokyo, Japan). Relative levels of immunostaining were evaluated by three independent observers, and this was repeated at least four times. 

### 2.8. Apoptosis Assay 

The level of testicular apoptosis-related DNA fragmentation was evaluated by TUNEL assay by using a commercially TUNEL kit (Servicebio, Wuhan, China). The sections of 4-μm paraffin tissue specimens were serially sectioned, and the standard method was employed according to the manufacturer’s instructions. The DAPI agent was used to visualize the apoptotic germ cells, and sections were observed under a light microscope. The cells in the testes exhibiting green nuclear staining were considered positive for nuclear DNA fragmentation. Data collected using a Nikon LSM 700 system (Nikon, Tokyo, Japan) and Image-Pro Plus6.0 software (Media Cybernetics, Maryland, USA). Data then analyzed by SPSS 25.0. (Beijing Wangshu Co., Ltd, China). Ten independent fields of vision were chosen as representative fields in each group mouse.

### 2.9. Serum Testosterone Measurement

The levels of serum testosterone (T) of PND35 mice in each group were measured by commercial radio immunoassay (RIA) kits (Nanjing Jiancheng Bioengineering Institute, Nanjing, China) according to the manufacturer’s instructions. The sensitivity of the assay was below 1.0 pg/ml. The intra- and inter-assay variation coefficients were both below 15%.

### 2.10. RNA Extraction and Real-Time PCR

Total RNA was isolated from mice testes at five weeks of age with Trizol® reagent (Vazyme Biotech, Nanjing, China) according to the manufacturer’s protocol. Further reversed and transcribed to generate cDNA using the Revert Aid First Strand cDNA Synthesis Kit (K1622, Thermo Fisher Scientific, MA, USA) according to the manufacturer’s protocol. The expression levels of *GAPDH*, *ESR1*, *ESR2*, *CYP19A1*, *SOX9* and *BRD7* mRNA were measured using TaqMan gene expression assay with TaqMan probes (*GAPDH* NM_001289726.1, *ESR1* NM_001302533.1, *ESR2* NM_207707.1, *CYP19A1* NM_001348171.1, *SOX9* NM_011448.4 and *BRD7* NM_012047.2). The PCR was performed in an ABI-7500 real-time PCR system (Thermo, New York, NY, USA). Relative mRNA expression was analyzed with a cycle threshold (Ct) in the linear range of amplification using *GAPDH* as an internal control. (Primer sequences used for qPCR analysis were shown in Supplementary data Table S1).

### 2.11. Statistical Analysis

Statistical analyses were performed using GraphPad Prism 6 (Pactera, Beijing, China). Data were expressed as mean ± SEM. Our Data were analyzed by one-way ANOVA, with multiple comparisons among groups tested by Tukey’s range test and *p* < 0.05 was considered to be statistically significant.

## 3. Results

### 3.1. Growth Performance

**Figure 1:**
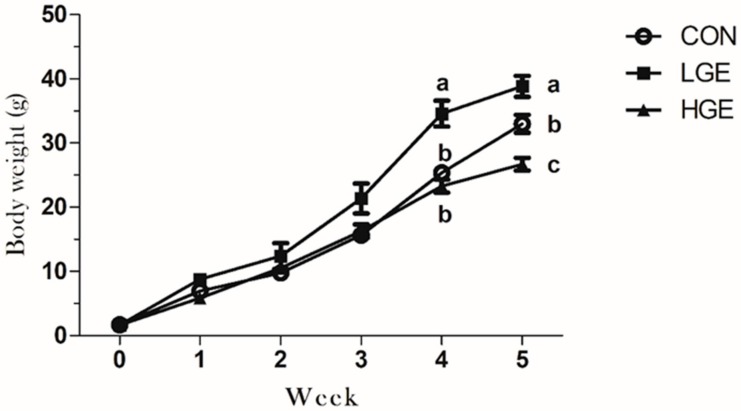
The effects of dietary GEN on male mice body weight. Data are shown from the time point of the newborn (0 week) to PND 35 (5 weeks) weekly. Each value represents the mean ± SEM (n = 7). Different labels indicate significant (*p* < 0.05) differences among groups.

The body weights of male pups were recorded weekly, and their mean values are presented in Figure 1. Generally, the body weight of the LGE group was significantly higher (*p* < 0.001) as compared to those of the controls (CON) on the fourth week and fifth week. However, the reduction (*p* < 0.001) of body weight in the HGE group (23.05%) was statistically significant compared with the CON group on the fifth week (Table 2), suggesting that dietary GEN might enhance the growth of mice in the LGE group, while HGE treatment could inhibit the growth of mice.

### 3.2. Serum Concentrations of Testosterone

**Figure 2:**
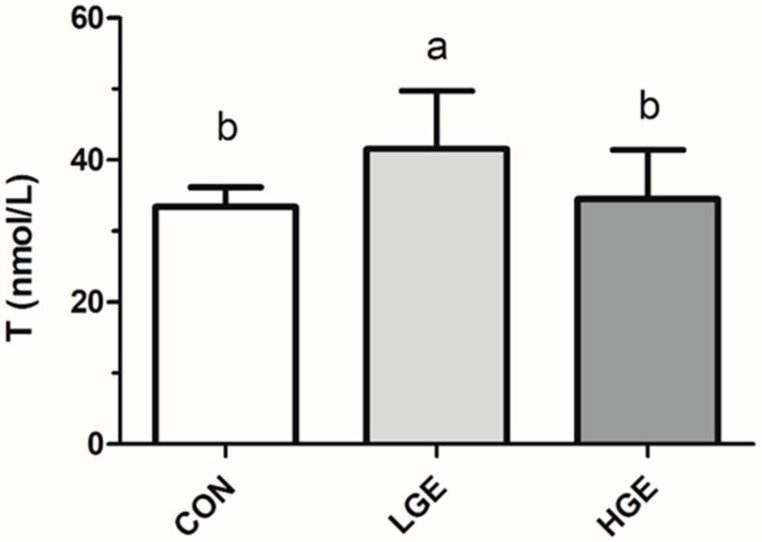
Serum concentrations of testosterone in mice. Each value represents the mean ± SEM (n = 7). Different letters (a–c) within a column represent significantly (*p* < 0.05) differences among groups, respectively.

We determined the serum testosterone (T) levels of experimental mice. LGE treatment significantly increased (*p* < 0.05) the level of T compared with the CON group. However, the level of T the HGE group did not show a statistical difference compared with the CON group (Figure 2).

### 3.3. Testis Histology

To determine the effect of different dose GEN on the testicular development, we evaluated the testis weight, the diameter of seminiferous tubules, heights of seminiferous epithelial and sperm abnormality rate of male mice at PND35. The testis weight of mice in the LGE group increased significantly (*p* < 0.001) compared with the CON group (Table 2). However, the testis weight of mice in the HGE group was lower (*p* < 0.001) than the CON group (Table 2). Similarly, the testis size (the diameter of seminiferous tubules) of mice in the HGE group was smaller (*p* < 0.001) than that in the CON group (Table 2). The height of seminiferous epithelium of mice in the LGE group was higher than that in the CON group (*p* < 0.05), while the height of epithelium in the HGE group was significantly decreased (*p* < 0.05) compared with the CON group (Table 2).

### 3.4. Effects of GEN Exposure on ESR1, ESR2, CYP19A1, SOX9 and BRD7 Expression in the Testis of Mice at PND35

**Figure 3:**
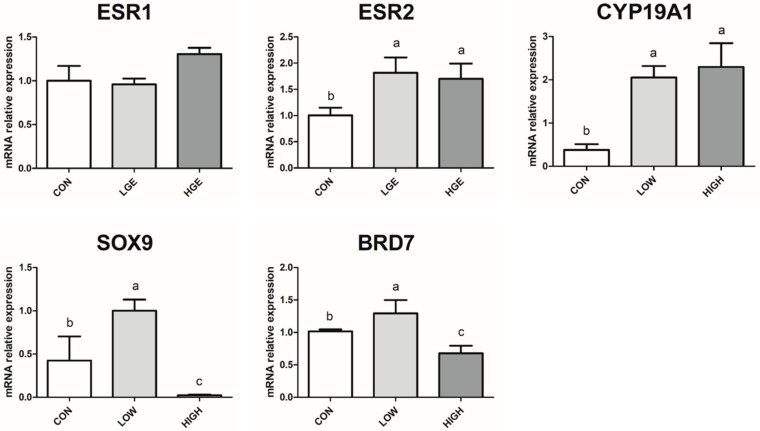
Effects of dietary GEN on reproduction-related gene expression in testis of mice at PND35. Each value represents the mean ± SEM (n = 7). Different letters (a–c) within a column represent significantly (*p* < 0.05) differences among groups, respectively.

To explore the role of different dose of dietary GEN in germ cell growth in testis, some reproduction-related genes were detected by qR-PCR analysis (Figure 3). *ESR1* and *ESR2* were estrogen receptors, which could mediate estrogen actions in testis. The mRNA expression of *ESR1* did not show a significant difference over the groups (*p* = 0.34). However, the level of *ESR2* expression was significantly increased (*p* < 0.05) after LGE and HGE treatment. These might reveal a new mediator of estrogen action in mice after dietary GEN administration. Furthermore, in order to clarify the effect of different dose of dietary GEN to testosterone levels and the spermatogenesis of the testis, *CYP19A1*, *SOX9* and *BRD7* were evaluated. Our results showed that the mRNA expression of *CYP19A1* in the LGE and HGE group were higher than the CON group (*p* < 0.001). Similarly, as Figure 3 shows, the mRNA expression of *SOX9* and *BRD7* in the LGE group were higher (*p* < 0.001) than that in the CON group. While the mRNA expressions of *SOX9* and *BRD7* decreased (*p* < 0.001) significantly in the HGE group when compared with the CON group (Figure 3). Therefore, we concluded that poor *SOX9* expression induced by HGE accounted for the testis growth deficiency.

### 3.5. PCNA Detection, TUNEL Assay and Testis Scoring

**Figure 4:**
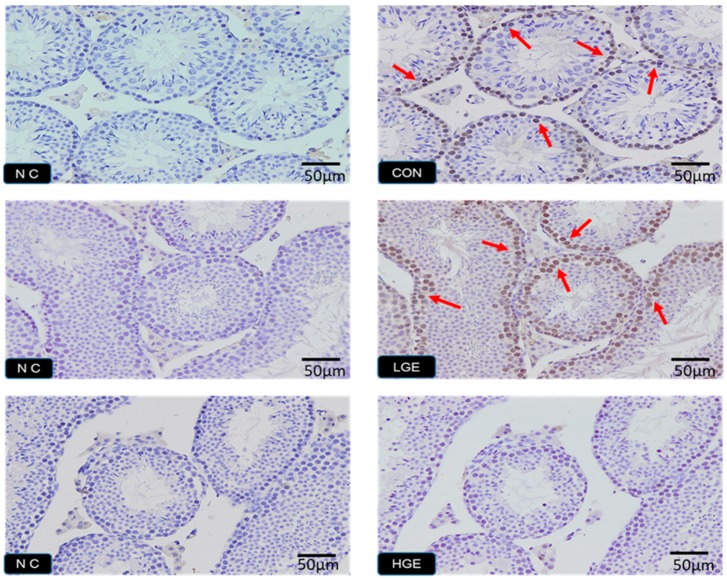
PCNA-positive germ cells of mice from GEN treatment. “NC” means negativehentrol. “CON, LGE and HGE” represent the control group, 40mg/kg GEN of the dietary group, and 800mg/kg of the dietary group, respectively. The magnification is 20×, OLYMPUS *BX51*. Positive germ cells are marked by red arrows.

**Figure 5:**
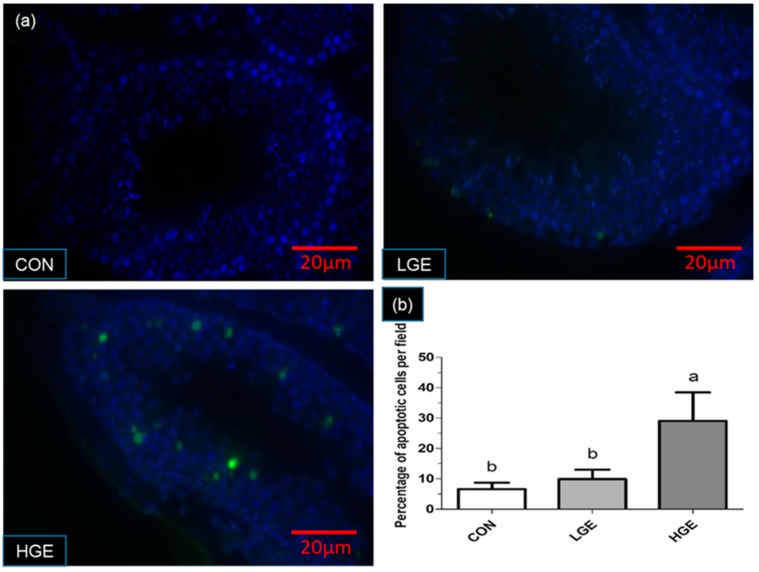
GEN-induced apoptosis of germ cells among the groups. (**a**) The figures of apoptotic cells in a different group; (**b**) The result of apoptotic cells detected by using Nikon ECLIPSE 80i. Each value represents the mean ± SEM (n = 7). 10 random fields of seminiferous tubules for each mouse were checked. Different labels indicate significant (*p* < 0.05) differences among groups. Scale bar = 20 μ m.

**Figure 6:**
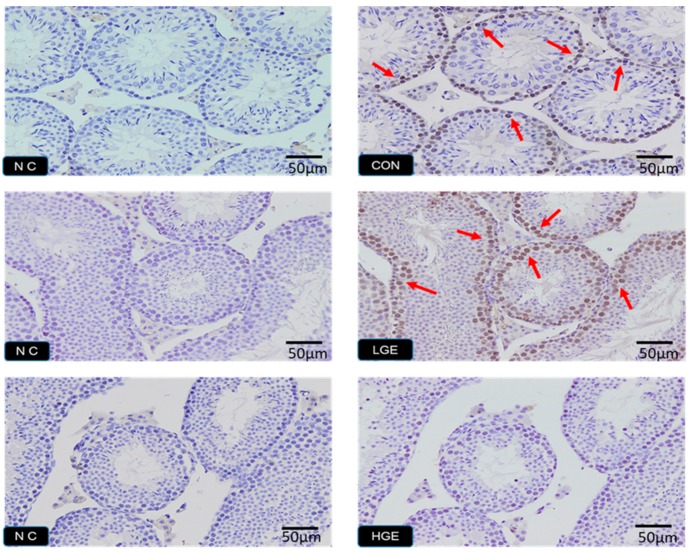
Effects of dietary GEN on the histoarchitecture of seminiferous tubules at PND 35 in mice. Three-group sections of tissue specimens were counterstained with hematoxylin and eosin. Figures were employed by a systematic method of light microscopic examination on Nikon ECLIPSE 80i photomicroscope. Each figure represents the most frequent structural changes. “CON, LGE and HGE” represent the control group, 40mg/kg GEN of the dietary group, and 800mg/kg of the dietary group, respectively. The magnification of the first line is 10 ×, and the second line figure is under 20× magnification.

**Figure 7:**
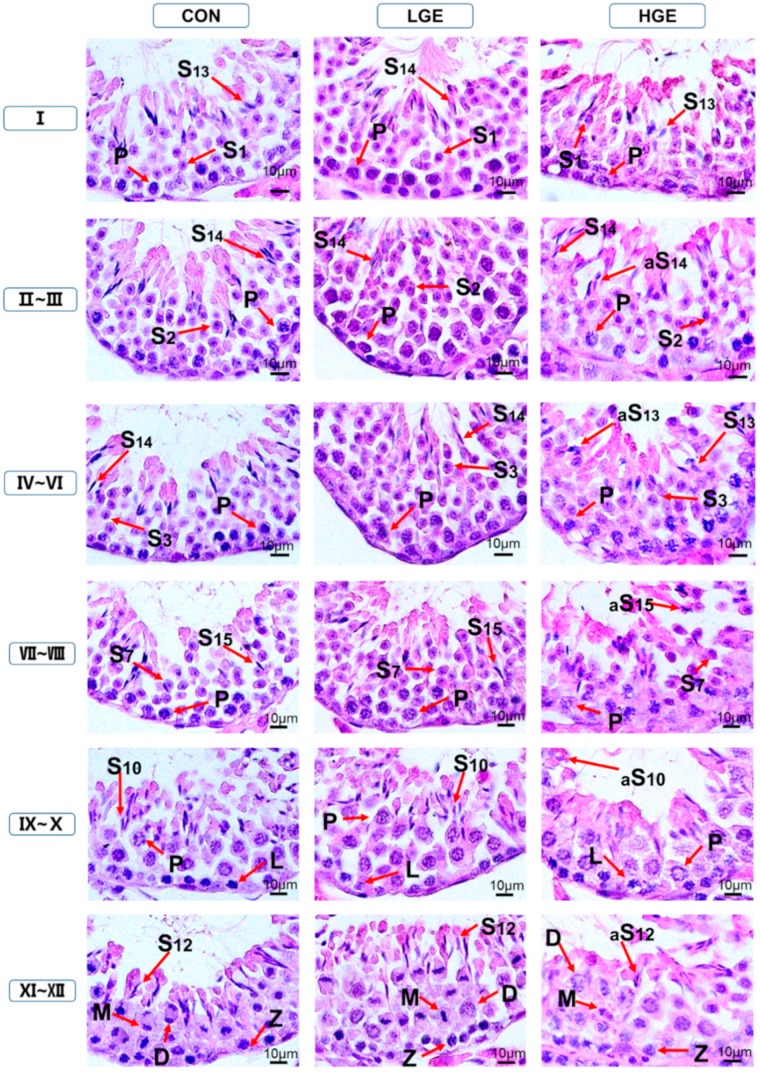
Dietary GEN increases abnormal spermatids in the testis. Hematoxylin and eosin-stained images of testes of GEN -treated mice at the age of five weeks. The images were evaluated using Nikon LSM 700 photomicroscope by a systematic method of light microscopic examination under 100× magnification with oil immersion. The number of abnormal spermatids, shown in Figure 3b. The images in each column represent the different experimental groups, including control (CON), low dose of GEN (LGE) and high dose of GEN (HGE). These figures show the histopathological changes at diverse stages in the cycle of spermatogenesis in the seminiferous tubule. These stages are marked at the left side of the figure and are denoted basing on the staging method defined for laboratory mouse [43,44]. Different abbreviated arrows and markings indicate the following: Z, zygotene spermatocyte; P, pachytene spermatocyte; D, diplotene spermatocytes; M, meiotic germ cell; S1–16, Step 1–16 spermatids. Different markings inside the images were inserted through Adobe Photoshop CS5 and bars are 10 μm in size.

The PCNA expression in the testes was detected by IHC. The weak expression of PCNA was observed in the CON group (Figure 4). More positive brownish yellow granules, including spermatogonium, spermatocytes and spermatids, were observed in the LGE group compared with the CON group. However, none of the PCNA-positive germ cells in the seminiferous tubules of the high group was observed (Figure 4). As shown in Figure 5, the TUNEL assay was employed to reveal the apoptosis process in spermatogenic cells. The number of apoptotic germ cells in the group was significantly increased (*p* < 0.05) compared with the CON group. In addition, We used spermatogenesis scoring method according to our recent study (Table 1). The result of testicular scoring in the LGE group was higher (*p* < 0.05) than that in the CON group (Table 2), which indicated a thriving testicular development of mice in the LGE group. Whereas, the score of testes the HGE group was much lower (*p* < 0.05) than the CON group (Table 2), which demonstrated poor spermatogenesis in HGE group. In addition, the features of each group are shown in Figure 6. Compared with the CON group, germ cells of mice in the LGE group during multiple stages of spermatogenesis, ranging from the spermatogonium to the spermatozoa stage developed well (Figure 6). Meanwhile, the height of the germinal epithelium of mice in the LGE group was increased significantly (*p* < 0.05) compared to the CON group (Table 2). However, the height of seminiferous epithelium in the HGE group decreased significantly (*p* < 0.05), (Table 2). Furthermore, severe destruction of the germinal epithelium and poor spermatogenesis in the testes were observed after HGE treatment. Multiple stages of germ cells of mice with HGE treatment developed loosely and un-synchronously (Figure 6). Figures and characters of spermatogenesis processing (Stage I-XII) in each group are shown in Figure 7. Transmission electron microscopy (TEM) was carried to evaluate the development of germ cells. More abnormal spermatids in the testis of the HGE group were observed (Result was presented in Supplementary data Figure S1). TEM examination results showed that the percentage of abnormal germ cells in the HGE group increased significantly (*p* < 0.001) compared with CON group (Table 2).

## 4. Discussion

The growth performance of pups could be affected when dietary exposure to GEN. Transgenerational effects, including epigenetic changes induced by maternal factors, lead to developmental changes in offspring [45,46]. GEN, an important member of the multifarious group of phytoestrogens, which affect growth performance, osteoporosis, and metabolic syndromes. Several lines of evidence have presented that GEN exerts different effects on male reproduction ability, which depends on the dose and species [39]. However, the role of GEN exposure on pups during the fetal period has not been adequately evaluated, especially on the reproductive system. It is reported that the body weight and food intake of rats were reported to increase after oral 300  mg/kg dietary GEN [47]. However, some study suggested that high dose of GEN (300 mg/kg) treatment could inhibit the growth of pups compared with a low dose of GEN (60 mg/kg) [48]. In this study, the body weight of male offspring decreased when both maternal and pups were supplemented with 800 mg/kg GEN. Previous studies revealed that the same result after oral GEN administration (50 mg/kg) [49]. Similarly, dietary GEN (40 mg/kg) treatment in the current study increased the body weight of offspring mice. Therefore, our results suggested that dietary GEN treatment can alter the growth behavior of offspring mice in a dosage-dependent manner. 

Apart from body performance, GEN might have an effect on testicular development. It revealed that changes in steroidogenesis could be dose-dependent after GEN administration [50]. There are data in the literature on reproductive system that GEN consumption decreases the testicular size and spermatogenesis [39,51]. In the present study, the HGE treatment (800 mg/kg) decreased the weight and size of the testis as well. Some studies also revealed that decline in the weight of testes inhibited from low body weight [49]. We supposed that reducing body weight might be the reason for the decline of testis weight. Meanwhile, we found that the spermatogonia development and the germ cell layers were decreased by HGE treatment. In the present study, the number of spermatocytes in each seminiferous tubule was decreased, which indicated poor proliferation of the spermatogonium and spermatocytes in the HGE group. Therefore, defective development of testis is due to the higher apoptosis ratio of germ cells after HGE administration. Interesting, LGE treatment (40 mg/kg) increased the testis weight of mice. We speculated that LGE treatment might give a positive effect of StAR expression and eventually exerted prospective proliferation of germ cell.

GEN may regulate the secretion of testosterone and eventually affect the growth of testis. Sexual hormones, including testosterone, are indispensable for the process of spermatogenesis in the testis. GEN can affect testicular development by modulating steroidal hormone receptors [52]. As we all know, testosterone plays a pivotal role in the regulation of structure development of testis [53]. Whereas, Zhang found that GEN could alter sex steroid hormone levels by modulating steroidogenic enzymes activity [48]. In particular, testosterone levels were reported to reduce after high dietary GEN (over 100 mg/kg) administration [54,55]. However, the level of testosterone of mice in the LGE group was higher than the CON group. A good arrangement of Sertoli cells and perfect spermatogenesis were observed, which may relate to a higher concentration of testosterone in the LGE group. Therefore, our data suggested that low dose of GEN treatment promotes testicular growth by increasing the secretion of testosterone and positively regulates testicular functions of male pups. Whereas, 800 mg/kg of GEN treatment may inhibit the spermatogenesis process according to the size and weight of testis, even if the concentration of testosterone did not show any significant changes.

There are differences in the expression of estrogen receptors and the aromatase. Since the main role of GEN involves its interaction with estrogen receptors, the estrogenic effect is commonly reported in the investigations of GEN [52,56]. The current study showed that LGE treatment exerts estrogenic activity and promotes spermatogenesis in PND35 male mice, while HGE treatment causes incomplete spermatogenesis. It is revealed that GEN administration can affect the development of steroidogenic capacity by upregulating steroidogenic enzyme expression [54,57]. Aromatase, encoded by *CYP19A1* gene, is a key factor in the biosynthesis of estrogens, predominantly estradiol-17 β [29,30]. Furthermore, testosterone is converted to estradiol via aromatase [58]. A higher level of *CYP19A1* expressions observed in GEN treatment group (LGE and HGE group), which indicated that endogenous estrogens maybe effective negative feedback to the hypothalamus-pituitary-gonad axis. *ESR1* and *ESR2* were detected for the physiological effects of estrogen, which is traditionally mediated by receptors ER, including ERα and ERβ [59]. Surprisingly, there are differences in the expression between *ESR1* and *ESR2*. In particular, a higher level of *ESR2* expression in the LGE and HGE group, but not RES1 compared with the CON group, which indicated that GEN might exert its function via *ESR2* receptor. The previous study also reported that *ESR2* has a greater affinity than *ESR1* [60,61]. Therefore, GEN seems to exert its estrogenic function via regulating some estrogen-related gene expression, and eventually affect the testicular development. 

Apart from affecting testosterone level, GEN may also participate in gene expression, which could regulate the spermatogenesis process. *SOX9*, a biomarker of the Sertoli cell, is the essential and indispensable regulator for survival and proliferation of germ cells [62]. *SOX9* is involved in the initiation and maintenance of Sertoli cell differentiation [63]. Moreover, Sertoli cells facilitate the development of pluripotent primordial germ cells into spermatogonia, which support the spermatogenesis throughout the lifespan of mice [31]. Some study also reported that differentiation of germline stem cells via actions of Sertoli cell, which could be inhibited by the *SOX9* expression [31,62]. The diameter of seminiferous was significantly decreased in the HGE group. Meanwhile, the height of seminiferous epithelium was significantly decreased, and none of the PCNA-positive germ cells was observed in the HGE group. Lower *SOX9* expression indicated dysfunction of Sertoli cells, which may be responsible for infertility of mice in the HGE group. However, the testis weight of mice in the LGE group was increased, and the volume of seminiferous tubule was expanded, which enhance the process of spermatogenesis. Meanwhile, plenty of PCNA-positive germ cells were observed in the LGE group. *BRD7* could regulate the proliferation of germ cells throughout the spermatogenesis of male mice. Moreover, mice knockout of *BRD7* resulted in infertility and spermatogenesis defects [32]. In this experiment, low expression of *BRD7* was observed in the HGE group. In addition, incomplete spermatogenesis was also observed in morphological figures in our study. Thus, downregulation of *BRD7* expression will be the result of testicular development inhibition in continuous exposure mice to GEN supplementations from the fetal period. 

## 5. Conclusions

In summary, this experimental model of mice exposed to GEN during the fetal and neonatal period brings to light the controversial effects on the testicular development of male offspring. HGE treatment could cause testicular problems of the pups by reducing the proliferation of germ cells and inhibiting the *SOX9* expression. Furthermore, HGE treatment may induce incomplete spermatogenesis and infertility, which associated with higher apoptosis ratio of germ cells. However, LGE treatment could promote the spermatogenesis of male offspring by reducing the apoptosis of the germ cells. Moreover, LGE treatment could positively regulate the expression of *SOX9* for testicular development. Therefore, we suggest that risk to reproductive health should be carefully considered, especially GEN is an easily accessible soy product with higher participation in daily life.

## Figures and Tables

**Table 1:** Criteria of spermatogenesis scoring method.

Score	Criteria of Scoring Method
5	Complete spermatogenesis and perfect tubules
4	Spermatozoa present with disorganized spermatogenesis
3	No spermatozoa, but long spermatids present
2	No long spermatids present, but round spermatids present
1	No spermatids, but spermatocytes present
0	Only spermatogonia present

**Table 2:** Effect of different dose of dietary GEN on testis weight and behavior data of PND 35 male mice.

Index	CON	LGE	HGE	*p*-Value
Body weight (g)	33.84 ± 0.63 ^b^	37.07 ± 0.75 ^a^	26.04 ± 0.65 ^c^	<0.001
Testis weight (g)	0.098 ± 0.003 ^b^	0.120 ± 0.006 ^a^	0.074 ± 0.003 ^c^	<0.05
Epididymis (mg)	0.031 ± 0.002	0.032 ± 0.001	0.027 ± 0.002	0.15
Seminiferous epithelium height (μm)	60.98 ± 3.52 ^b^	89.34 ± 4.51 ^a^	38.08 ± 4.86 ^c^	<0.05
Seminiferous tubules Diameter (μm)	177.83 ± 2.41 ^a^	180.80 ± 2.08 ^a^	153.92 ± 5.98 ^b^	<0.001
Abnormal spermatids (%)	8.1 ± 2.55 ^b^	9.28 ± 3.09 ^b^	50.20 ± 7.17 ^a^	<0.001
Testes scoring	3.48 ± 1.07 ^b^	4.35 ± 0.11 ^a^	2.88 ± 0.26 ^c^	<0.05

Values are expressed as mean ± SEM (n = 7). “CON” means control group. “LGE” and “HGE” represent 40mg/kg and 800mg/kg GEN respectively. In each column, different labels indicate significant differences among groups for each parameter at *p* < 0.05.

## Supplementary Materials

The following are available online at https://www.mdpi.com/2076-2615/10/3/377/s1, Figure S1: Figure S1. Transmission electron microscopy (TEM) of the testis images from GEN treatment., Table S1: Primer sequences used for qPCR analysis.

## Abbreviations:

GEN	Genistein
CON	Control group
LGE	Low dose of genistein diet
HE	High dose of genistein diet
PD	Pregnancy days
PND	Post-Natal days
T	Testosterone
*ESR1*	Estrogen receptor 1
*ESR2*	Estrogen receptor 2
*CYP19A1*	Cytochrome P450 family 19 subfamily A member 1
*SOX9*	Sry-related HMG box 9
*BRD7*	Bromodomain-containing protein 7
PCNA	Proliferating cell nuclear antigen
HE	Hematoxylin-eosin staining
