# Supplementary material for: Oral Exposure to Genistein during Conception and Lactation Period Affects the Testicular Development of Male Offspring Mice
Source: Animals (Basel). 2020 Feb 26;10(3):377. doi: 10.3390/ani10030377 (PMC7143625; doi:10.3390/ani10030377)
Supplement: Supplementary file 1 [file animals-10-00377-s001.pdf]

Table S1. Primer sequences used for qPCR analysis.

Gene	Genebank number	Sequence of nucleotide (5′-3′)	Product length (bp)
GAPDH	NM_001289726.1	Forward: GGCAAATTCAACGGCACAGT<br>Reverse: CTCGTGGTTCACACCCATCA	255
ESR1	NM_001302533.1	Forward: AGGTCCACCTTCTCGAGTGT<br>Reverse: TCCCAGTTGTCTGAAGTCCTTG	180
ESR2	NM_207707.1	Forward: GACGAAGAGTGCTGTCCCAA<br>Reverse: TCAGCTTCCGGCTACTCTCT	209
BRD7	NM_012047.2	Forward: ATCGTGGGAAACAGCTTCGT<br>Reverse: AGCACCTCCAAGTTCAGAATGA	150
SOX9	NM_011448.4	Forward: GTGCAAGCTGGCAAAGTTGA<br>Reverse: TGCTCAGTTCACCGATGTCC	106
CYP19A1	NM_001348171.1	Forward: GGGGGCATACACATCTGGAG<br>Reverse: TCTTGCGCTATTTGGCCTGA	77

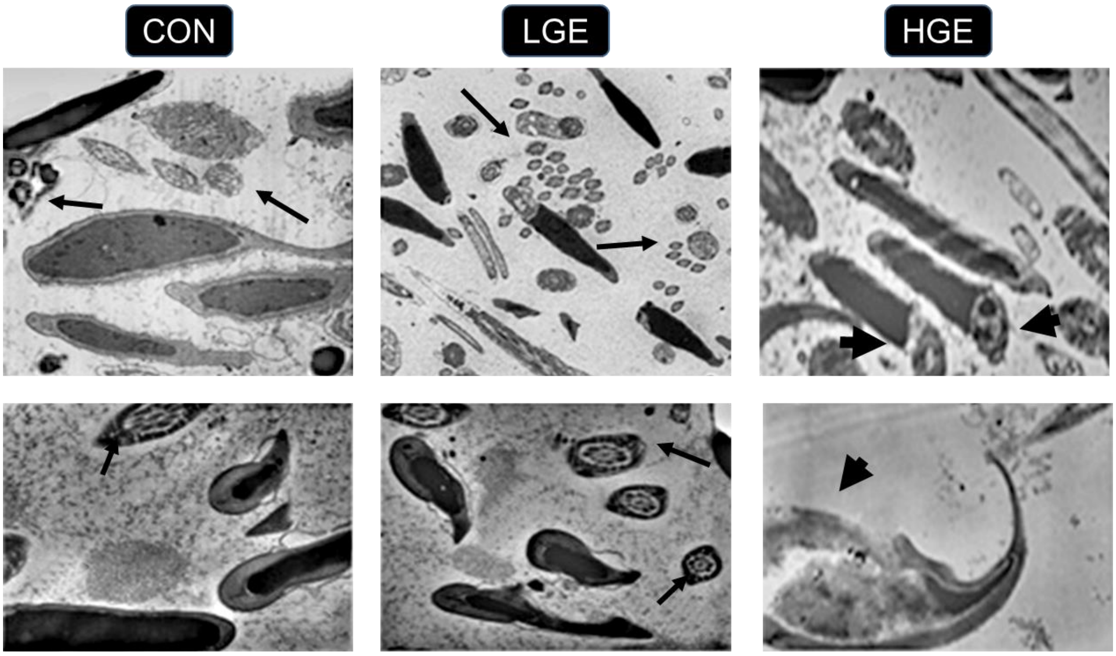

Figure S1. Transmission electron microscopy (TEM) of the testis images from GEN treatment. The arrows indicate the sperm and the arrowheads indicate abnormal nuclei or acrosome. Magnification 20000×.
